# Can childcare work be designed to promote moderate and vigorous physical activity, cardiorespiratory fitness and health? Study protocol for the Goldilocks-childcare randomised controlled trial

**DOI:** 10.1186/s12889-020-8291-y

**Published:** 2020-02-17

**Authors:** Mark Lidegaard, Anders Fritz Lerche, Pernille Kold Munch, Kathrine Greby Schmidt, Charlotte Lund Rasmussen, Charlotte Diana Nørregaard Rasmussen, Svend Erik Mathiassen, Leon Straker, Andreas Holtermann

**Affiliations:** 1 National Research Centre for the Working Environment, Copenhagen, Denmark; 2 Novo Nordisk A/S, Novo Nordisk Health & Safety, Bagsværd, Denmark; 3 Department of Public Health, Section of Social Medicine, University of Copenhagen, Copenhagen, Denmark; 4 Department of Occupational Health Sciences and Psychology, Centre for Musculoskeletal Research, University of Gävle, Gävle, Sweden; 5 School of Physiotherapy and Exercise Science, Curtin University, Perth, Western Australia Australia; 6 Institute of Sports Science and Clinical Biomechanics, University of Southern Denmark, Odense, Denmark

**Keywords:** Ergonomics, Workplace intervention, Cardiometabolic fitness, Physical activity, Physical work demand, Productive work, Sedentary behaviour, Workplace health promotion

## Abstract

**Background:**

Despite extensive efforts, issues like obesity and poor physical capacity remain challenges for a healthy work life in several occupations. The Goldilocks work principle offers a new approach, encouraging design of productive work to promote physical capacity and health. This paper presents the protocol for the Goldilocks-childcare study, a randomised controlled intervention trial aiming to evaluate the effectiveness of implementing the Goldilocks work principle in childcare. The primary aim of the intervention is to increase time in moderate to vigorous physical activity (MVPA) by having the childcare workers act as active role models for children in daily playful physical activities, and thereby improve cardiorespiratory fitness and health of the workers.

**Methods:**

The study is a cluster-randomised trial with a usual-practice wait-list control group. The 10-week intervention consists of two phases. In the first, the childcare workers will participate in two participatory workshops aiming to a) develop playful physical activities (‘Goldilocks-games’) for children in which childcare workers participate as active role models at MVPA intensity, and b) develop action plans for implementation of the Goldilocks-games in daily work routines. In the second phase, childcare institutions will implement the Goldilocks-games. The primary outcome is working time spent in MVPA, and secondary outcomes are cardiorespiratory fitness, sleeping heart rate, perceived need for recovery, and productivity. Primary outcome and process evaluation will be based on direct measurements of physical activity and heart rate, determination of cardiorespiratory fitness, and questionnaires.

**Discussion:**

If proven effective, the Goldilocks work principle has a large potential for promoting sustainable health and working lives of childcare workers.

**Trial registration:**

ISRCTN, ISRCTN15644757, Registered 25th December 2019

## Background

Despite extensive efforts to promote occupational health, several occupations still face considerable challenges in achieving a long, healthy, and sustainable work life for those employed. Examples of these challenges are the increased occurrence of obesity in the working population [1], and a considerable proportion of workers not having the physical capacity required for performing their main work tasks [2]. Both issues are associated with social inequality in health [3], and particularly pronounced in an ageing working population [4].

The dominant approach to prevent work-related disorders has consisted of reducing the physical activity demands at work [5]. Accordingly, the intensity of occupational physical activity has been minimised in many occupations, most often by increasing the amount of sedentary time [6]. To compensate for insufficient physical activity at work, numerous workplace health promotion initiatives have then attempted to improve employees’ physical capacity and health by offering physical exercise at the workplace [7, 8]. However, these initiatives have not been successful in reaching the employees most in need [9–11]. Also, workplace physical exercise programmes are often not appealing to employers, since they require time away from productive work, and thus being a costly initiative. Therefore, both minimising physical activity and introducing exercising during working hours are inadequate to solve the abovementioned challenges in occupational health [12].

As an alternative, the Goldilocks work principle has been proposed [12, 13], aiming to design productive work in a way that promotes physical capacity and health without compromising productivity [12, 13]. Building on work physiology fundamentals [14], the Goldilocks work principle seeks to achieve a ‘just right’ balance between physical activity demands and recovery at work, so that a training effect can be obtained from the work per se, leading to better health [12]. However, the effectiveness of the Goldilocks work principle remains to be evaluated in randomised controlled trials.

Childcare workers generally report a high prevalence of poor general health, physical work exertion, musculoskeletal pain, and sickness absence [15–17]. Moreover, childcare workers have been shown to spend only a small amount of work time in moderate to vigorous physical activity (MVPA) [18]. In a pilot study conducted among childcare workers, we found workers were sitting extensively and had minimal work time in MVPA. Because MVPA is well documented to improve cardiorespiratory fitness and health [19], we believe that an increase in MVPA in the daily routines of childcare workers could have a great potential to lead to better health and more sustainable work.

An important general aim in childcare is to encourage children to be more physically active [20, 21]. Therefore, by acting as active role models in daily playful physical activities (termed ‘Goldilocks-games’) together with the children, the childcare workers may be able to achieve sufficient daily work time in MVPA to improve their own cardiorespiratory fitness and health. However, this has not been evaluated in a randomised controlled trial among childcare workers.

The aim of this protocol paper is to describe the design, implementation, and evaluation of the participatory randomised controlled trial ‘Goldilocks-childcare’, which seeks to increase the childcare workers’ productive work time spent in MVPA, and thus their cardiorespiratory fitness and health.

## Methods/ design

### Data protection, ethical approval, and trial registration

The National Research Centre for the Working Environment has an institutional agreement with the Danish Data Protection Agency about procedures to treat confidential data (journal number 2015-41-4232), e.g. by securing data at a protected drive with limited access, and by anonymizing all individual data.

The Danish National Committee on Biomedical Research Ethics (The local ethical committee of Frederiksberg and Copenhagen) has evaluated a description of the study and concluded that, according to Danish law as defined in Committee Act § 2 and § 1, the intervention described should not be further reported to the local ethics committee (Ref number: H-18041423).

The study is registered in the International Standard Registered Clinical/soCial sTudy Number (ISRCTN) registry (ISRCTN15644757). The protocol conforms to the Standard Protocol Items: Recommendations for Interventional Trials (SPIRIT) 2013 statement [22] and the Template for Intervention Description and Replication (TIDieR) checklist [23]. The reporting of the study will follow the Consolidated Standards of Reporting Trials (CONSORT) 2010 statement [24].

### Study design

**Fig. 1:**
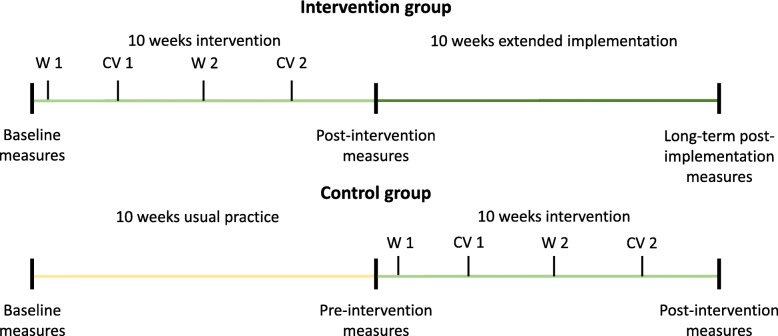
Study design. W1 = A two-and-a-half-hours workshop (Workshop 1) explaining the overall concept of the Goldilocks work principle. CV 1 = Consultant visit at the childcare institutions performed by the work environment consultants. W2 = A one-and-a-half-hour follow-up workshop (Workshop 2). CV2 = a consultation phone call with a member of the Trio 2 weeks after Workshop 2

Figure 1 provides an overview of the study design. The study will use a cluster-randomised design with a usual-practice wait-list control group. As the study is an organisational intervention in a work place setting, individual randomisation is neither possible nor appropriate [25]. Thus, each participating institution will form a cluster. A wait-list design (offering the intervention to the intervention and, eventually, even to the control groups) was chosen in an attempt to minimise any potential lack of commitment from an institution acting as control [26]. Thus, we will randomly assign the participating childcare institutions to, i) the intervention group immediately receiving the intervention after the baseline measurements or, ii) the control group continuing usual practice for 10 weeks after the baseline measurements and then performing the intervention. Each intervention period will last 10 weeks. After the 10 weeks intervention for the intervention group, they will be encouraged to continue with Goldilocks-games without further support from the research team.

Recruitment of childcare institutions opened in March 2019 and will continue until the sufficient number of childcare institutions required for the trial are enrolled. Recruitment of participants at the participating childcare institutions will open in January 2020. The intervention will start in January 2020 with the participants enrolled in the childcare institutions recruited first. The last childcare institutions enrolled in the trial will start the intervention by September 2020.

### Study population

The childcare institutions are recruited from the greater Copenhagen area in collaboration with employer organisations, unions, and local government municipalities. In order to be eligible for participation, the childcare institution should employ a minimum of nine childcare workers.

As the intervention is organisational, all employees within the participating childcare institutions will take part in the intervention activities, and all childcare workers will be eligible for participation in the trial evaluation. Since this participation is voluntary, the childcare workers will, prior to entering the trial, be provided with information about the trial, asked if they agree to participate, and if so, asked to sign an informed consent form.

### Randomisation and blinding

Cluster-randomisation will be used with each childcare institution constituting a cluster, in order to avoid contamination within an institution between participating and non-participating workers. Participating childcare institutions will be randomised to either intervention or usual practice (i.e. wait-list control) arms of the trial. This randomisation will be conducted upon enrolment of each participating childcare institution into the study. The randomisation sequence has been developed using the statistical software R [27].

Because of the time needed for the childcare institutions to plan their participation in the trial, we need to inform them about their allocation to intervention or control group before the baseline data collection. Moreover, due to the nature of the trial, it will not be possible to blind neither the researchers nor the participating employees as regards whether a particular institution is subject to the intervention or not. However, allocation concealment will be maintained throughout the study, and all researchers conducting the randomisation, statistical analysis, and evaluation will be blinded.

### Intervention

The overall study idea was developed in collaboration between the researchers and work environment consultants (Physiotherapists and Occupational therapists) from the Work Environment Consultancy of Copenhagen Municipality (WECoCM), based on the Goldilocks work principle [12].

To ensure that the intervention is relevant for, tailored to, and closely integrated with pedagogical teaching aims as well as feasible for the childcare institutions, the intervention will apply a participatory approach. The Goldilocks work principle for the intervention content and implementation is explained and modified on the basis of a dialog with stakeholders related to childcare (e.g. employer organisations and unions, practitioners in childcare, work environment consultants), of observations of childcare work, and of a dialog with managers and employees in childcare institutions.

Further, the manager, a union representative, and an occupational health and safety representative (collectively referred to as the Trio) from each of the participating childcare institutions will attend a workshop. The workshop will outline the Goldilocks work principle for all institutions and aim to facilitate planning and management support to the implementation and evaluation of the intervention. The Trio will then be involved in planning and tailoring the intervention process to their own childcare institution. Thus, the Trio is responsible for outlining the pedagogical focus, rostering and practical planning at their institutions. Involving the Trio at an early stage will enhance the likelihood of organisational buy-in and the possibility for introducing organisational changes.

To facilitate development and implementation of the Goldilocks-games, we will conduct a proof of concept study in a few childcare institutions. The proof of concept study will focus on development and evaluation of feasible Goldilocks-games that can increase the occurrence of MVPA among the childcare workers. Experiences from this proof of concept study will be applied in the first workshop at each institution participating in the randomised controlled trial.

**Fig. 2:**
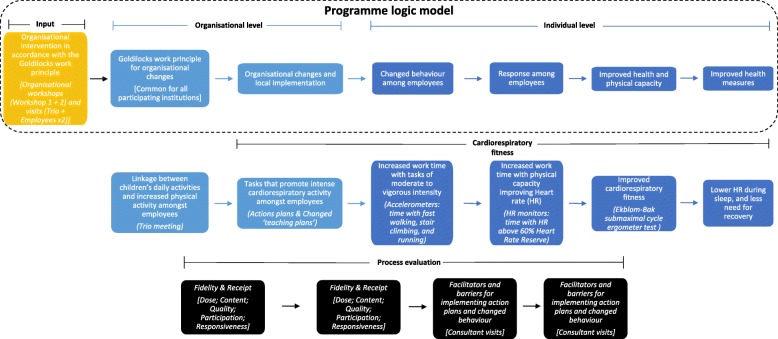
Programme logic model of the Goldilocks-childcare randomised controlled trial. The dashed oblong represents the overall programme logic for the Goldilocks work principle. The golden box represents the input, the light blue boxes the intended steps at an organisational level, and the dark blue boxes the intended steps at an individual level. The black boxes illustrate the process evaluation. The middle row (Cardiorespiratory fitness) shows how the intervention will expectedly lead to improved cardiorespiratory fitness. The bottom row (Process evaluation) illustrates how the ‘black box’ between each step of the programme will be evaluated. Heart rate (HR) reserve is defined as the difference between the estimated maximal heart rate and the heart rate during sleep

A programme logic model for the Goldilocks work principle was developed (Fig. 2). The programme logic model provides a schematic overview of the steps from introducing the Goldilocks work intervention to the effects on cardiorespiratory fitness and health of the childcare workers. In addition, the programme model assists in guiding the effect and process evaluation.

### Delivery of intervention

Work environment consultants (Physiotherapists and Occupational therapists) from the Work Environment Consultancy of Copenhagen Municipality (WECoCM) will deliver the intervention components (workshops and visits). In order to ensure a consistent delivery across the participating institutions, we have developed an intervention protocol describing all intervention components.

### Intervention content

**Fig. 3:**
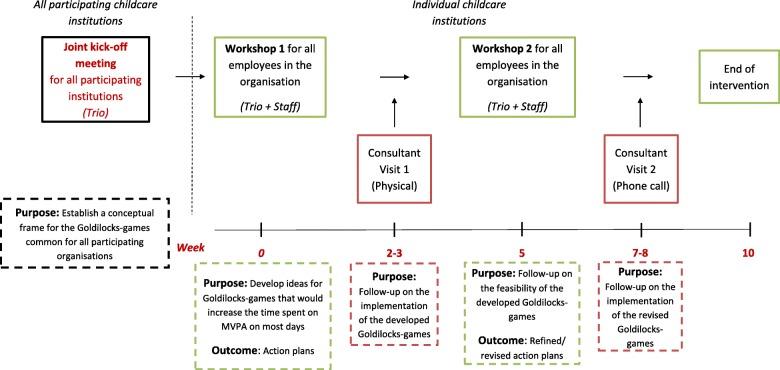
Overview of intervention components. Trio = the group at each of the participating institutions consisting of a manager, a union representative, and an occupational health and safety representative. MVPA = Moderate to Vigorous Physical Activity

Figure 3 provides an overview of the intervention components. At each individual childcare institution, the intervention will be initiated by a two-and-a-half-hours workshop (Workshop 1) during a regular staff meeting. At Workshop 1, the work environment consultants will inform the participating Trio and childcare workers about the overall concept of the Goldilocks work principle, facilitate that the participants develop tailored Goldilocks-games compliant with their pedagogical teaching goals, and finally develop specific action plans allocating responsibilities for implementation of the Goldilocks-games in their daily routines and schedules. Information regarding whether the Goldilocks-games were conducted as planned will be collected.

After three to four weeks of the intervention period, the work environment consultants will conduct a one-and-a-half-hour follow-up workshop (Workshop 2) with the participants at each individual childcare institution. The aims will be to i) evaluate the implementation of the Goldilocks-games, ii) facilitate sustainability of well-functioning Goldilocks-games, and iii) modify those Goldilocks-games, which are not working as intended. Moreover, in order to facilitate implementation of the Goldilocks-games, the work environment consultants will make a consultation visit at the childcare institution 2 weeks after Workshop 1 and have a consultation phone call with a member of the Trio 2 weeks after Workshop 2.

### Control group

Institutions in this group serve as waitlist controls. Thus, institutions in the control group will continue their usual practice for the first 10 weeks, while institutions in the intervention group implement the intervention. Following the 10-week period, control group institutions will implement the intervention in the same manner as those in the intervention group.

### Data collection

Data will be collected at three time points: At baseline; at a 10-week follow-up (Immediate post-intervention for intervention group and Pre-intervention for control group); and at a 20-week follow-up (Long-term post-intervention for intervention groups and Immediate post-intervention for control group). Baseline and immediate post-intervention data collection will consist of i) an electronic questionnaire through a link provided in a text message to the participants, ii) anthropometric measurements, iii) testing of cardiorespiratory fitness, and iv) wearable sensor technical measurements of physical activity and heart rate. At the long-term post-intervention follow-up, data collection will include only a modified questionnaire.

### Questionnaire

The questionnaires include a combination of items to assess the following descriptive factors of the study population, as well as potential confounders, and beneficial and adverse effects i) sociodemographic factors, i.e. age; sex; ethnicity; length of service; job title; and weekly working hours, ii) health and behaviours, i.e. musculoskeletal pain and pain-related work interference [28]; medicine use; smoking; general health [29, 30]; self-efficacy [31]; well-being [32]; and sleep behaviour [33], iii) self-rated physical capacity: cardiorespiratory fitness and muscle strength [34], iv) stress [35], and v) work environment factors, i.e. perceived physical exertion during work [36]; productivity [37]; psychosocial work environment [38]; single item work ability [39]; short-version perceived need for recovery [40]; and sickness absence and presenteeism [41, 42].

Need for recovery and productivity will act as secondary outcomes. Need for recovery will be determined using a short, three-item version: ‘At the end of my work day I am exhausted’; ‘I find it hard to show interest in other people, when I have just come home from work’; and ‘It takes me over an hour before I am fully recovered after a work day’. All items have five response categories: ‘Never’; ‘Rarely’; ‘Some of the Time’; ‘Most of the Time’; and ‘Always’ [40]. Productivity will be determined using one item: ‘On a scale from 0 to 10, where 0 is the worst job performance anyone could have at your job and 10 is the performance of a top worker, how would you rate your overall job performance on the days you worked during the past 4 weeks (28 days)?’ [47].

### Anthropometric measures

We will measure height (Seca 213; Seca GmbH, Hamburg, Germany) and weight (BC-418 MA body composition analyzer; Tanita, Tokyo, Japan), and calculate the body mass index (body weight [kg]/ (body height squared [m^2^])). In addition, we will determine fat percentage (BC-418 MA body composition analyzer; Tanita, Tokyo, Japan) and resting blood pressure (Omron M3 or Omron M6 Comfort; Omron Corporation, Kyoto, Japan).

### Physical activity and heart rate

#### Physical activity type and body postures

Physical activity type (i.e. moving, walking, running, bicycling, climbing stairs), body position (i.e. sitting, and standing), and number of steps will be measured using a thigh-worn AX3 accelerometer (3-Axis Logging Accelerometer; Axivity Ltd., Newcastle upon Tyne, UK) and processed using the validated Acti4 software [43–45].

The AX3 accelerometer generates measurements of linear acceleration in three dimensions with a dynamic range of ± 8 G, sampled with a precision of 13 bits at a sampling rate of 25 Hz. The AX3 accelerometers are initialised prior to recording, and data will be downloaded using the manufacturer’s software (OMGUI Version 1.0.0.30; Axivity Ltd).

One AX3 accelerometer will be mounted on the right thigh at the most muscular part of the quadriceps femoris, midway on the line between the anterior inferior iliac spine and the top of the patella [44]. The AX3 accelerometer will be mounted on the skin with adhesive tape (Hair-Set double-sided adhesive tape; 3 M Company, Maplewood, MN, USA) and secured with transparent adhesive film (Opsite Flexifix; Smith & Nephew plc, London, UK). We will ask the participants to wear the accelerometer around the clock during five working days.

#### Heart rate and heart rate variability measures

We will measure heart rate and heart rate variability using a Firstbeat Bodyguard 2 monitor (Firstbeat Technologies Ltd., Jyväskylä, Finland). The monitor measures the electrocardiogram at a sampling frequency of 1000 Hz, and the signal is processed to identify R-spikes and subsequently R-R intervals. The monitor has been validated for long-term measurements of heart rate in free living [46].

The Firstbeat Bodyguard 2 will be mounted with Ag/AgCl pre-gelled electrodes (Ambu WhiteSensor CMM-00-S/30; Ambu A/S, Ballerup, Denmark) below the right clavicle and at the left rib cage. We will download data from Firstbeat Bodyguard 2 using the manufacturer’s software (Firstbeat Uploader Version 3.1.2.0; Firstbeat Technologies Ltd., Jyväskylä, Finland). As for the accelerometer, we will ask the participants to wear the heart rate sensor around the clock during five working days.

### Cardiorespiratory fitness

Cardiorespiratory fitness will be assessed using the Ekblom-Bak submaximal test [47] performed on a cycle ergometer (Monark AB, Varberg, Sweden). The Ekblom-Bak test estimates cardiorespiratory fitness (VO_2max_) based on the difference in heart rate between an initial low standard workload and a subsequent higher ‘final’ workload. The test has shown good validity in a wide population range (Women: 21–86 years old with a VO_2max_ range of 19–62 ml/ min/kg; Men: 20–84 years old with a VO_2max_ range of 24–76 ml/min/kg [47]).

The test is initiated by having participants performing a standard workload of 60 revolutions/ minute at a resistance of 0.5 kp for 4 min. Heart rate is measured four times during the last minute (3:15, 3:30, 3:45, and 4:00) and the average of these four measurements is used as the initial heart rate. Subsequently, resistance is increased in steps with the aim of reaching a perceived exertion rating of approximately 14 on the Borg RPE-scale [36], and a heart rate between 120 and 150, or 110 and 140 beats/ minute for participants younger or older than 50 years, respectively. Perceived exertion is rated after 2 min at each step. If below 10 or 12, resistance is incrementally increased by 1 kp or 0.5 kp, respectively, for younger and older participants. When reaching the target exertion and heart rate, four measurements are collected during the last minute, with the average heart rate of these four measurements providing the final heart rate [47]. Cardiorespiratory fitness is calculated from equations described in [47].

### Primary and secondary outcomes

Differences between the intervention and the control groups in changes from baseline to 10-week follow-up will be evaluated for all primary and secondary outcomes. The primary outcome is the relative work time spent in MVPA, as determined by either heart rate (i.e. ≥60% of heart rate reserve) or accelerometer recordings (i.e. fast walking (≥130 steps/ minute), running, or stair climbing).

**Fig. 4:**
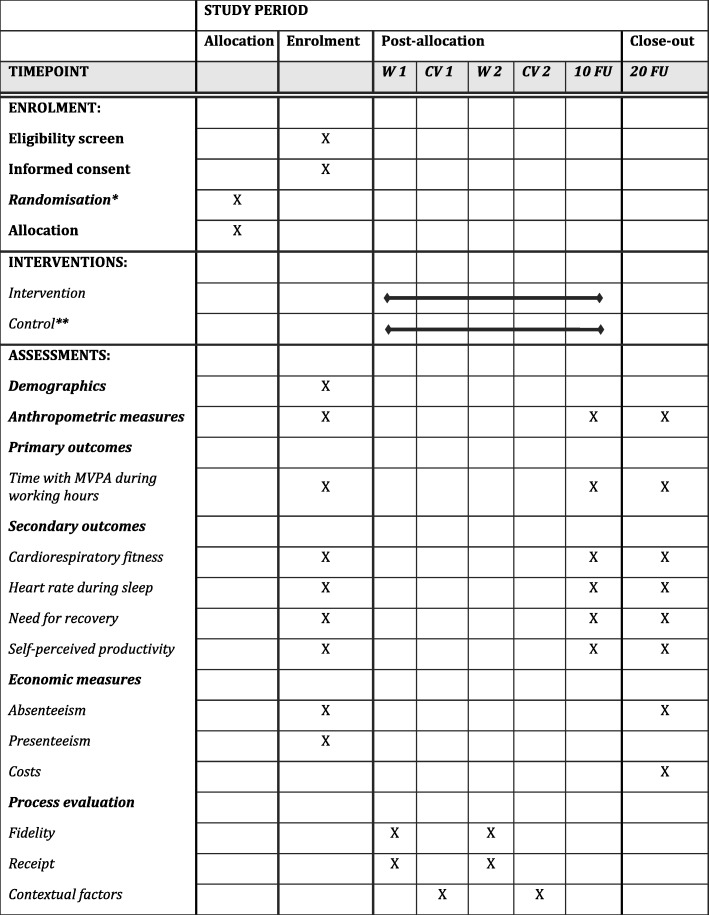
Standard Protocol Items: Recommendation for Interventional Trials (SPIRIT) schedule of enrolment, interventions, and assessments. W = Workshop; CV = Consultant visit; FU = Follow up; MVPA = Moderate to Vigorous Physical Activity. *Randomisation carried out at childcare institution level before baseline measurements. **The study uses a wait-list design; control group institutions will receive the intervention after the intervention group

Furthermore, the study has four secondary outcomes: i) cardiorespiratory fitness, ii) resting heart rate during sleep, iii) perceived need for recovery; and iv) self-reported productivity. Figure 4 shows the SPIRIT schedule of enrolment, interventions, and assessments [22].

### Economic evaluation

The economic evaluation aims to determine the cost-effectiveness of the intervention in terms of cost (from the employer’s perspective) per increment in work time spent in MVPA. Costs of the intervention will include costs related to implementation and operation, specifically:

#### Staff time

Participation in intervention activities for workers (workshops, and kick-off meeting for the project), and Trio (workshops, kick-off meeting for the project, and time spent on planning the logistics of implementing the intervention) will be assessed based on registration of attendance. Costs for participants and supervisors will subsequently be estimated based on their average yearly gross salaries, including overhead.

#### Consultant time

Time spent on delivery of the intervention (workshops, kick-off meeting, and workplace visits) will be assessed by asking the work environment consultants how many hours they spent on these activities, including preparations. The number of working hours will be valued by using their hourly fee, including overhead.

#### Consumables

Materials, such as printouts and posters, as well as fruit/snacks/coffee consumed at meetings will be noted. Costs will be valued using invoices.

For the control group, only costs related to participation in the joint kick-off meeting will be included.

The total intervention costs for the employer will be estimated and compared between the intervention and control group. The incremental cost-effectiveness ratio will be calculated by dividing the mean difference in costs (incremental cost) between both groups by the difference in effects (incremental effect) on the primary outcome measure.

In addition, sickness absence will be measured by questionnaire from the participants in the control and intervention groups at baseline and 10-week follow-up.

Costs associated with absenteeism will be estimated using the Friction-Cost approach [48]. A friction period of 4 weeks will be assumed, as the Danish social security system takes over costs after 4 weeks of sickness absence. Furthermore, an appropriate elasticity factor will be used. Health-related productivity losses will be valued using gross yearly salaries of the participants converted to a daily cost based on assumed numbers of working days per year.

### Process evaluation

Before the intervention starts, we will collect information addressing facilities at the childcare institutions, including possible areas for the Goldilocks-games and accessibility to these areas. Further, we will collect information about readiness for change among employees at the childcare institutions.

To assess the extent to which the intervention is implemented as intended, a process evaluation will be conducted. The process evaluation will follow the principles described by Steckler and Linnan (2002), and Ferm and colleagues (2018) [49, 50]. The process evaluation will assess how the intervention was delivered (Fidelity) and received (Receipt) [49].

Fidelity includes three measures i) Dose (the number of intervention components delivered); ii) Content (if the components are delivered in accordance with the workshop manual); and iii) Quality (the self-rated performance of the deliverer). Receipt includes two measures i) Participation (number of participants attending the two workshops); and ii) Responsiveness (satisfaction and motivation among the participants). Following each workshop, the work environment consultants will assess dose, content, quality, and participation in a customised questionnaire. Responsiveness will be assessed by questionnaire to the participants following the intervention.

Additionally, we will collect information regarding facilitators and barriers for implementing the intervention through semi-structured interviews with managers during the consultant visits. These interviews will provide information on contextual factors for each of the childcare institutions that could have influenced the implementation or the effect of the intervention, e.g. occurrence of major organisational changes during the intervention period, or concurrent activities with a likely impact.

### Power calculation

We estimated the number of participants to be included in the trial based on a statistical power analysis of the primary outcome using clustered parallel groups with before-and-after measures to determine the design effect. The power calculation was based on data from a larger sample (*N* = 167) of childcare workers in Copenhagen in a previous trial (ISRCTN10928313) [51]. In this sample, work time spent with a heart rate reserve (HRR) ≥60% was, on average, 1.24 min/day with a standard deviation (SD) between subjects of 2.90. The power calculation was done after processing data according to the principles of compositional data analysis (CoDA) [52, 53], where work time spent at HRR ≥60% is expressed relative to time spent at HRR < 60% using isometric log-ratios (ilr) [54–56]. Expressed as an ilr, the average relative work time spent at HRR ≥60% was − 4.35 (SD = 1.10).

Based on these transformed data, we will need an estimated total of 132 participants (corresponding to approximately 14 childcare institution clusters shared between the intervention and wait-list groups) to be able to detect (at *p* < 0.05) a 5 min/day increase in relative work time spent at HRR ≥60% with a power of 0.80, an estimated intra-cluster correlation coefficient (ICC) of 0.05, a fixed cluster size of 10, and an assumed drop-out rate of 30%.

### Statistical analysis

Evaluation of intervention effectiveness on the primary and secondary outcomes as well as the cost-effectiveness will be based on multilevel models, taking into account that the study design implies repeated measurements within each participant [57]. Conclusions about the effectiveness of the intervention with respect to the primary and secondary outcomes will be based on the group effect and its 95% confidence interval. The 95% confidence intervals for the incremental cost-effectiveness ratio will be estimated using bootstrapping (1000 bootstrap samples with replacement) [58]. All analyses will be performed according to the intention-to-treat principle [59]. Potential confounding factors (e.g. baseline differences between the intervention and control group in factors like age and BMI) will be adjusted for in the statistical analyses.

## Discussion

This will be the first randomised controlled trial to evaluate the effectiveness of the Goldilocks work principle in terms of increased work time in MVPA and better cardiorespiratory fitness for workers. If successful, the study will demonstrate that the Goldilocks work principle has a potential to improve health and physical capacity among workers while performing productive work. A scaling of the Goldilocks work principle to other occupations and countries could have a large impact on general health and social justice of working populations [12].

### Strengths and limitations of the study

A methodological strength of the study is the use of a cluster randomised design, which minimises the risk of contamination within and between institutions in the intervention and control groups. Another methodological strength is the application of a systematic participatory approach involving the end users throughout the entire process. We will collect experiences and information during the process of developing and tailoring the intervention, and this can likely benefit the present as well as future studies. Even the use of wearable accelerometers and heart rate monitors for measuring the primary outcomes is a strength. By using wearable sensors rather than self-reported methods to record the primary outcome, bias introduced by self-reports, e.g. due to lack of blinding of participants or inaccurate perception [60], is considered minimal.

In addition, utilizing CoDA is a strength of this study, since it allows the co-dependency between work time spent in different behaviour types (sedentary behaviour; light physical activity; MVPA) to be taken into account. Thus, the use of CoDA represents an approach that gives a better understanding of the potential effect of an intervention from a whole-workday perspective than if each behaviour is analysed as an independent factor [56, 61]. Furthermore, conducting a process evaluation is a strength of this study. The process evaluation provides an opportunity for thoroughly evaluating the implementation of the intervention and identify why the intervention may or may not be effective.

The main limitation of the study is the lack of allocation concealment, which introduces the risk of selection bias. This is, however, inevitable since the study design requires the participating childcare institutions to make organisational changes that build on a participatory approach. Thus, the participating childcare institutions need to be informed of their allocation in advance, in order to facilitate their introduction of organisational changes as well as allowing them adequate time for preparing and initiating any required logistics, e.g. staffing issues or equipment. Also, blinding the participants is, for natural reasons, not possible. This is a common issue in participatory interventions, and it may introduce a risk of unintended effects, e.g. a possible placebo change in the selected outcome(s), or a Hawthorne effect [62]. However, we will try to minimise both of these limitations. All participants will, eventually, receive the intervention, participants are only informed about when the intervention will be implemented at their institution, and not whether they are allocated to an intervention or a control group in the actual intervention project. Thus, the lack of blinding is expected to have no or minimal influence on the results of the study.

### Trial status

The study is ongoing. Recruitment of childcare institutions opened in March 2019 and will continue until all childcare institutions required for the trial are enrolled, planned to be September 2020. The intervention will start at the first enrolled childcare institutions in January 2020. For the last childcare institutions enrolled in the trial, the intervention is planned to start September 2020. We will finalize the intervention for the last group of childcare institutions in December 2020.

## Data Availability

Not applicable.

## Abbreviations

CoDA: Compositional data analysis
CONSORT: Consolidated standards of reporting trials
HR _sleep_: Sleeping heart rate
HR: Heart rate
HR_max_: Estimated maximal heart rate
HRR: Heart rate reserve
ilr: Isometric log-ratio
ISRCTN: International Standard Registered Clinical/soCial sTudy Number
MVPA: Moderate to vigorous physical activity
SD: Standard deviation
SPIRIT: Standard protocol items: recommendations for interventional trials
TIDieR: Template for intervention description and replication
Trio: Collective term for group consisting of a manager, a union representative, and an occupational health and safety representative
VO_2max_: Maximal oxygen consumption
WECoCM: The Work Environment Consultancy of Copenhagen Municipality
